# COVID-19 in Iran: the Good, the Bad, and the Ugly Strategies for Preparedness – A Report From the Field

**DOI:** 10.1017/dmp.2020.261

**Published:** 2020-09-27

**Authors:** Morteza Arab-Zozani, Djavad Ghoddoosi-Nejad

**Affiliations:** Social Determinants of Health Research Center, Birjand University of Medical Sciences, Birjan, Iran; Social Determinants of Health Research Center, Faculty of Health, Department of Public Health, Birjand University of Medical Sciences, Birjand, Iran

**Keywords:** emergency preparedness, emergency responders, public policy

## Abstract

The emergence of coronavirus disease 2019 (COVID-19), a novel unknown virus that is challenging whole countries all over the world, has prompted different strategies from various governments. Iran, as one of the first countries to experience the onset of the virus outbreak, made and implemented some policies that should be assessed, so that lessons may be learned for the future. Although some negative actions and policies, such as delays in cancellation of international flights especially from China, not taking the disease seriously and comparing it with seasonal influenza, and the like, are hard to ignore, some impressive actions are also vividly clear. Policies, such as social distancing, dramatically increasing social awareness about preventive actions in terms of public health, and using masks and hand washing, were cost-effective policies that resulted in successful control of the virus in the first onset. While some quite clearly ineffective decisions were made by Iranian authorities, the huge catastrophic effect of sanctions cannot be forgotten. Possibly in level situations with similar countries, Iran will have far better results regarding preparedness for future pandemics like COVID-19.

Having officially been labeled a pandemic, the coronavirus disease 2019 (COVID-19) has so far devastated over 213 countries and territories around the world and 2 international conveyances.^1,2^ This challenging, historic, and memorable phenomenon the world is experiencing is teaching us an extremely tough but useful lesson in the way we deal with potential disasters going forward, by making a case for the most effective policy-making and management practices.^3^

The importance of policy analysis, during times of crisis and especially with pandemics, is highly valuable. Taking the wrong steps in combination with inadequate policies has the power to endanger the lives of many. Considering the level of contagiousness, as well as its international reach, this virus has the potential to affect lives not just in 1 country but the population of the entire world.^3,4^

Emerging from Wuhan, China, in early December of 2019, the virus has spread all over the world. Iran was one of the first countries affected by the virus, and although the authorities’ initial reaction at the beginning of the epidemic was to “keep calm, and ignore the seriousness of the situation by comparing it to seasonal influenza,” they subsequently had to change their position and consider tougher controls in the cities, as the course of the virus outbreak seemed to be out of control.^5^

Iran’s health system, under overwhelming pressure because of COVID-19, is experiencing a unique situation in which very novel and yet valuable lessons can be learned for the years to come.^6^ In this regard, we are going to assess Iran’s steps and actions to assess these proceedings as well as outline and evaluate its impact.

## Narrative

On a time-based approach, the first and foremost event was a delay in canceling all flights from China, while other countries simultaneously shut down all air travel from that region.^7^ This resulted in an increased risk of spreading the disease by infected people entering the country. Authorities are speculating that it was the students from Wuhan who were the main reason for spreading the virus into Guilan Province, a northern province of Iran. Clearly, given the high contagious characteristic of the virus in combination with communications and travel (air transport in particular), this had a potential to easily accelerate the spread of such diseases.^7,8^

The next event that increased the rate of disease prevalence is the demonstration on Independence Day, February 11th - 22 Bahman in the Persian calendar, while China had officially announced infection by the virus and were taking the measures of disallowing all travel from the region; this might have flattened the curve of the disease brought by the travelers who had not yet shown any symptoms. Numerous studies have shown that close contact, as well as disregarding the importance of social distancing, could be one of the factors causing the virus to spread rapidly.^3,5,8,9^ Due to the political importance of this event and the lack of awareness of the disease within the country at the time, allowing such ceremonies to be held on a regular basis and with no distancing protocol could be another reason for a drastic increase of the spread of the disease in the country.

Moreover, despite the official announcement of the presence of the first infected patients with COVID-19, the decision to hold the parliament election turned out to be the third major factor in increasing the spread of the virus. Large gatherings of people in voting centers, despite all cautions about the COVID-19, took place, and this led to a further spread of the virus.

The fourth, and some would say the most important factor, was deciding not to quarantine Qom, the first city in which the virus was officially announced. The presence of a large number of religious school students from diverse ranges of countries, especially from China, made Qom the first city with a widespread prevalence of COVID-19. Given that the majority of students from different cities in Iran are present in Qom as well, it was not so hard to understand the virus would have travelled widely, with the city not being quarantined. While quarantine prevents the spread of contamination, it is one of the most primitive ways of controlling such diseases, inasmuch as in Wuhan, China, this strategy turned out to be very effective.^5^

Despite authorities’ primary denial of the COVID-19 incidence, the disease gradually was spreading to other cities. Consequently, with increasing concerns about the virus, schools and universities sent their students home. Delay in shutting the universities resulted in sending potentially contaminated students from big cities back to their homes, some of them small towns.

Continuous disregard of the seriousness of pandemic presence within the society was partially, however, due to the primitive approaches of social media. National TV called it less fatal than influenza, and having announced the decision to impose several days off work to the public, some people took the opportunity to go on trips, mostly to northern parts of the country, which had already been red-marked in terms of the virus epidemic. After that, in the very last hours of the Friday, the government changed the plan by suspending the days-off work policy, but it was clearly too late.

Concurrent with questionable actions of the public authorities, some opportunistic jobbers who claimed traditional medicine, they called it Islamic medicine, prescribed various alternative solutions that were in contrast with the practice of modern scientific medicine. This, alongside a bewilderment of public authorities, resulted in a confused population, which added to the ill handling of controlling the pandemic.

One thing about lack of collaboration in various sections was obvious. For example, while the Ministry of Health authorities did their best to inform the public about the disease by advising people to stay at home on national TV, just after the speech, the TV played an advertisement promoting a sale event for one of the biggest chain stores in Iran, which clearly contradicted the messages from health-related authorities, like they were living in a whole another country.

However, the effect of international sanctions on the economy of the country cannot be ignored.^6^ Lack of basic supplies, such as masks and general personal protective equipment (PPE), as well as inadequate numbers of beds per capita compared with other developed countries, caused a shock to the health system, particularly in hospital care and more specifically in intensive care unit (ICU) beds. Lack of medical equipment, such as computed tomography scanners and ventilators, forced healthcare managers to resort toward inevitable rationing. Not being able to screen everybody with symptoms due to limited availability of diagnostic kits was one of the main reasons for the World Health Organization (WHO) statement declaring that the official statistics in Iran are probably far less than the real figures.

On the bright side, participation and voluntary collaboration of people’s efforts to decontaminate public spaces, spreading information about the disease, and several tele-medical approaches for screening people, were positive steps in controlling the disease.

What is more, although during the New Year holiday (Nowruz) there were concerns about travelling to various provinces such as Isfahan, Yazd, and Fars, and at first no serious regulations were imposed as authorities resorted to advising the public, rather than enforcing the law, on the last days of the holidays, however, the government decided to put serious limitations in place, and although rather late, this might just be better late than never.

Another positive aspect of Iran’s authorities’ actions was focusing on primary healthcare policies that are proven to be more cost-effective than just clinical tertiary care. Increasing public knowledge, promoting their attitude,^10^ and also improving their practices resulted in a somehow successful management of the virus in the first onset.

Last but not least was the dedication and commitment of the personnel of the health-related centers, including primary, secondary, and tertiary levels of encountering the virus and infected people. Given the horrifying situations the virus can cause, these people proved their value, and it was one of the most memorable moments in the history of health care in Iran.

## DISCUSSION AND CONCLUSIONS

To summarize the above, it seems that, although some good actions are hard to ignore, in aggregate, Iran’s crisis management procedures, in terms of handling epidemics, are far from perfect. There is a lot to be learned from this event to better prepare for any similar events in the future. After all, the cruel heavy sanctions imposed on the government hurt the common people; its role should be considered when judging Iran’s policy-making decisions about managing COVID-19.
